# Prolonged Treatment with Propofol Transiently Impairs Proliferation but Not Survival of Rat Neural Progenitor Cells *In Vitro*

**DOI:** 10.1371/journal.pone.0158058

**Published:** 2016-07-05

**Authors:** Arvind Palanisamy, Matthew B. Friese, Emily Cotran, Ludde Moller, Justin D. Boyd, Gregory Crosby, Deborah J. Culley

**Affiliations:** 1 Department of Anesthesiology, Perioperative and Pain Medicine, Brigham & Women’s Hospital, Harvard Medical School, Boston, Massachusetts, United States of America; 2 Faculty of Pharmacy, Department of Pharmaceutical Biosciences, Uppsala University, Uppsala, Sweden; 3 Laboratory for Drug Discovery in Neurodegeneration (LDDN), Harvard NeuroDiscovery Center, Harvard Medical School, Cambridge, Massachusetts, United States of America; University of Pennsylvania, UNITED STATES

## Abstract

Neurocognitive dysfunction is common in survivors of intensive care. Prolonged sedation has been implicated but the mechanisms are unclear. Neurogenesis continues into adulthood and is implicated in learning. The neural progenitor cells (NPC) that drive neurogenesis have receptors for the major classes of sedatives used clinically, suggesting that interruption of neurogenesis may partly contribute to cognitive decline in ICU survivors. Using an in vitro system, we tested the hypothesis that prolonged exposure to propofol concentration- and duration-dependently kills or markedly decreases the proliferation of NPCs. NPCs isolated from embryonic day 14 Sprague-Dawley rat pups were exposed to 0, 2.5, or 5.0 μg/mL of propofol, concentrations consistent with deep clinical anesthesia, for either 4 or 24 hours. Cells were assayed for cell death and proliferation either immediately following propofol exposure or 24 hours later. NPC death and apoptosis were measured by propidium iodine staining and cleaved caspase-3 immunocytochemistry, respectively, while proliferation was measured by EdU incorporation. Staurosporine (1μM for 6h) was used as a positive control for cell death. Cells were analyzed with unbiased high-throughput immunocytochemistry. There was no cell death at either concentration of propofol or duration of exposure. Neither concentration of propofol impaired NPC proliferation when exposure lasted 4 h, but when exposure lasted 24 h, propofol had an anti-proliferative effect at both concentrations (P < 0.0001, propofol vs. control). However, this effect was transient; proliferation returned to baseline 24 h after discontinuation of propofol (P = 0.37, propofol vs. control). The transient but reversible suppression of NPC proliferation, absence of cytotoxicity, and negligible effect on the neural stem cell pool pool suggest that propofol, even in concentrations used for clinical anesthesia, has limited impact on neural progenitor cell biology.

## Introduction

Up to 30–50% of adult Intensive Care Unit (ICU) survivors suffer from long-term neurocognitive dysfunction, including impairment of attention, visual-spatial memory loss, and poor executive function, ultimately leading to long-lasting decreases in quality of life. [1–4] While there are undoubtedly numerous patient, disease, and treatment-related risk factors that predispose patients to the development of ICU cognitive impairment, the medications used for sedation have received a great deal of attention because they have profound CNS effects, are often administered for long periods, are implicated in neurotoxicity in some settings, and represent one of the few factors clinicians can readily modify.[3–9] Among these sedatives, gamma-amino butyric acid type A (GABA_A_) agonists such as propofol enjoy considerable popularity.[5–7, 9, 10]

Formation of new memories requires generation of new neurons from neural progenitor cells (NPC) and the subsequent integration of these newborn neurons into pre-existing neural circuitry.[11–13] This neurogenesis is robust during development but persists into adulthood in a few areas of the brain such as the olfactory bulb and hippocampal dentate gyrus, a region critical for learning.[14–17] In preclinical studies, genetic ablation of neural progenitor cells in adults causes defects in learning and memory, indicating that a healthy cohort of NPCs is important for maintenance of cognitive health throughout life. GABA and glutamate are important molecular drivers of these processes [18–24] and as such, prolonged stimulation of GABA_A_ receptors, as occurs during ICU sedation, may have deleterious consequences. For example, we among others have found that isoflurane, a prototypical inhalational agent that potentiates GABA_A_ receptors, decreases proliferation of NPC in a concentration-dependent manner, and depletes the NPC pool without causing NPC death.[25–28] However, much less information is available for propofol, and the limited evidence that is available pertains to its short-term use in the operating room rather than the ICU.[29–31] In the latter setting, sedation rather than anesthesia is the goal, so medications are administered at sub-anesthetic doses over a much longer time period (> 24h).

Propofol, a GABA_A_ agonist, is one of the most widely used sedatives in the ICU. Preclinical studies regarding propofol’s effects on NPCs are conflicting, with propofol reportedly either increasing, decreasing, or having no effect on the proliferation of NPCs in vivo or in adult-derived NPCs in culture.[30, 32–36] This variability may be due to differences in experimental paradigms related to the route, concentration, and duration of administration of propofol, as well as differences in the method of quantification of NPC proliferation. Here, we hypothesize that prolonged exposure to propofol concentration- or duration-dependently kills or markedly decreases the proliferation of NPCs. Because adult and embryonic NPCs are qualitatively similar,[37] we tested this hypothesis in embryonic rat NPCs with concentrations of propofol consistent with deep anesthesia, administered for 4 and 24h, and evaluated the effect of such treatment on NPC proliferation and survival. We found no evidence for propofol-induced NPC toxicity or apoptosis. However, prolonged treatment with propofol caused a duration-of-exposure dependent suppression of NPC proliferation but this effect fully resolved within 24 h.

## Materials and Methods

### Neural Progenitor Cell Harvest and Culture

All animal experiments were approved by the Harvard Medical Area Standing Committee on Animals (Boston, MA). Neural stem/progenitor cells were harvested from timed pregnant embryonic day 14 Sprague Dawley rats (Harlan Sprague Dawley, Indianapolis, IN) as previously described.[25] The pregnant dam was euthanized with a 5-min exposure to 100% CO_2_ prior to isolation of the NPCs from embryonic telencephalons. No other inhalational agent was used for harvest. We isolated neural progenitor cells (NPCs) from 16 pregnant dams. NPCs from all embryos in a particular litter were pooled as one biological unit and subjected to experiments after the 2^nd^ passage. We excluded 2 harvests because of suspected contamination. Each assay was repeated 3 times using independent biological samples from different dams, so each assay had N = 3 for biological replicates. NPCs were cultured in B27 medium which consists of Dulbecco's Modified Eagle Medium/F12 high glucose (Invitrogen), supplemented with glutamine (1:200, Invitrogen), Fungizone^®^ antimycotic (1:100, Invitrogen), penicillin-streptomycin (1:100, Invitrogen), B27 supplement without vitamin A (1:50, Invitrogen), and mitogenic growth factors FGF-2 and EGF (Peprotech) as previously described.[25] All experiments were performed 24 hours after the second passage to ensure consistency, which typically occurred on the 8th day in vitro (DIV). After the second passage, 10^4^ NPCs in 100μl of medium was added to each well of a 96-well poly-L-ornithine/laminin coated microplates (BD BioCoat, BD Biosciences, San Jose, CA) using a multichannel pipette (Eppendorf, Westbury, NY), and placed in a humidified cell culture incubator at 37°C with 5% CO_2_ overnight prior to treatment.

### Propofol Treatment

At the beginning of treatment, the medium from each well was removed and replaced with fresh B27 media containing 0.1% DMSO and 0 μg/mL (control), 2.5μg/mL, or 5.0μg/mL of propofol (2,6-diisopropylphenol, Acros Organics or Sigma-Aldrich). These concentrations were chosen to simulate predicted effect-site concentrations of propofol at loss of consciousness. [38–40] Furthermore, this concentration range is consistent with plasma propofol levels that are required to maintain sedation scores of 3–5 in mechanically ventilated patients. [41] NPCs were treated for either 4 or 24 hours, and were either fixed with 4% paraformaldehyde (PFA) at the end of treatment, or washed with phosphate-buffered saline (PBS) 5 times at the end of treatment, after which B27 media without propofol was added. The latter plates were placed in the incubator for 24 hours following which the NPCs were fixed with 4% PFA. Based on previous experience,[25, 42] we used 18 wells per treatment condition per time point for experiments to investigate cell death and proliferation as described below. NPCs from a single harvest were subjected to multiple experiments (cell death, EdU incorporation, cleaved caspase-3, etc.,) and experiments were repeated with NPCs from independent dams/harvests to generate a biological replicate of N = 3 per assay.

#### Propidium Iodide (PI) Staining

In plates assigned for quantification of cell death, 100μL of 1:100 propidium iodide (2mg/mL stock, Invitrogen) in B27 medium was added to each well after completion of treatment and allowed to incubate for 5 minutes prior to fixation with 4% PFA. We were unable to perform the lactate dehydrogenase (LDH) assay for cell death because of a significant concentration-dependent optical interaction with propofol.

#### EdU Incorporation Assay

Cell proliferation was determined with EdU incorporation using a commercially available kit according to the manufacturers’ instructions (Click-iT™ EdU Alexa Fluor^®^ High-Throughput Imaging (HCS) Assay, Invitrogen) as described previously.[25] Briefly, 20μM EdU in 100μL of B27 medium with growth factors was added to 18 wells per treatment condition of a 96-well plate (number based on previous experience with the assay). The final concentration of EdU was 10μM in each well. For the 4h exposure paradigm, EdU was added at the same time as treatment with propofol, but for the 24h group, EdU was added during the last 4 hours of treatment. This was necessary because of the length of the S-phase of the rodent NPC cycle. In the propofol withdrawal experiments, EdU was added during the last 4 h of the 24 h after treatment was discontinued (i.e., at 20 h after conclusion of treatment). NPCs were then fixed with 4% PFA in PBS for 15 minutes at which time the fixative was removed and the cells washed twice with 250μL PBS using an automated plate washer (Molecular Devices), and incubated in 100μL of 0.5% Triton^®^ X-100 in PBS for 20 min at room temperature. The cells were then washed twice and incubated with 100μL Click-iT™ reaction cocktail for 30 minutes at room temperature. The reaction cocktail was removed and the cells were washed twice with PBS and stored until fluorescence immunocytochemistry was performed for nestin, followed by staining with Hoechst 33342 to detect and label all nuclei to facilitate total cell counts. The nuclear intensity threshold for an EdU positive cell was defined by the intensity of EdU reactivity in NPCs from control wells.

#### Immunocytochemistry

After the EdU incorporation assay, cells were washed three times with PBS, and blocked with 100μL/well 3% BSA in PBS for 1 hour at room temperature followed by incubation with the primary antibodies nestin (Millipore, Billerica, MA, 1:500 dilution) and cleaved caspase-3 (CC3, Abcam Inc., Cambridge, MA, 1:500 dilution) overnight at 4°C. Nestin is widely used as a marker for stemness while CC3 is a marker of early apoptosis. The wells were then washed 3 times with PBS and species-matched secondary antibody (Alexa Fluor^®^ 488 or Alexa Fluor^®^ 555, 1:200, Invitrogen) was applied and incubated for 1 hour at room temperature. The wells were washed 3 times and then incubated with 100μL of 5 μg/mL Hoechst 33342 in PBS for 30 minutes at room temperature, washed three times with PBS, and stored in the dark at 4°C until image acquisition and analysis. NPCs treated with 1μM staurosporine for 6 hours served as a positive control for CC3 experiments. For PI and CC3 experiments, a nuclear/cellular intensity ratio greater than 2 or 2.5 indicated cell death or apoptosis, respectively.

### Image Acquisition and Analysis

#### Image Acquisition

Eight images (number based on previous experience with this system) were acquired per well using an IN Cell Analyzer 1000 (GE Healthcare, Piscataway, NJ) in an automated unbiased fashion as described previously. Because image acquisition is automated and large numbers of images can be acquired, cell selection bias is eliminated and the impact of experimental and biological variation is reduced. The parameters used for cellular identification were identical to those reported in our previous study with isoflurane.[25] For identification and analysis of EdU, PI and CC3 positive cells, we used a two-step filtering process. In the first step, nestin-negative cells were excluded; in the second, threshold setting was used to determine the number of EdU, PI, and CC3 positive cells. Thus, only nestin-reactive cells were analyzed and included in the final analysis. Imaging parameters were set based on the control wells stained with the fluorophore or antibody of interest and the parameters used to image all treated wells on the plate. In these experiments, 8 images were acquired per well with a 20X objective from 12–18 wells per treatment condition per assay per time. All experiments were repeated in at least three biologically independent NPC cultures.

#### Statistical Analysis

Data from each assay that passed equal variance testing were analyzed using 1-way ANOVA followed by Dunnett’s multiple comparison tests against control treatment at each time point. Data that did not pass equal variance testing were analyzed with Kruskal-Wallis 1-way ANOVA followed by Dunn’s testing for multiple comparisons. Data analysis was performed using Prism 5 for MAC OS X (Graphpad Software, Inc, La Jolla, CA) software and expressed as mean ± S.E.M; P ≤ 0.05 was accorded statistical significance.

## Results

**Fig 1:**
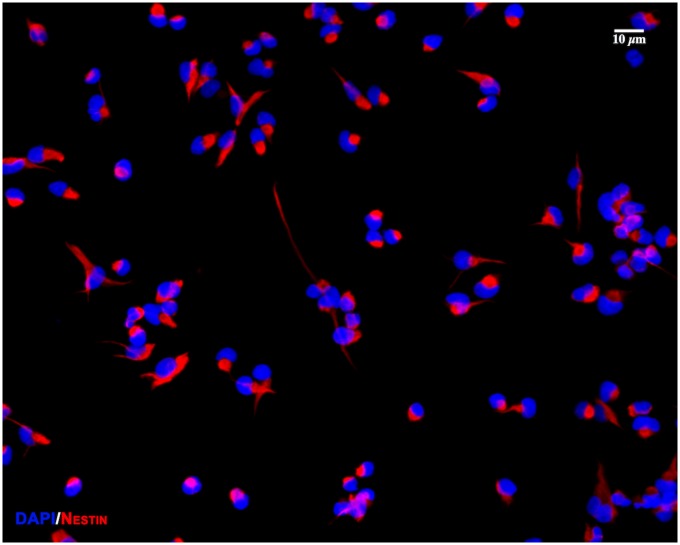
More than 96% of isolated cells express nestin. 5 x 10^4^ NPCs were seeded overnight on Corning^®^ BioCoat™ Poly-D-Lysine/Laminin 12 mm coverslips, permeabilized, and incubated overnight with 2μg/ml of mouse anti-nestin (EMD Millipore) and visualized with species-matched Alexa-Fluor^®^ 555 secondary antibody. A 20x image showing co-localization of nestin (red) with almost all of the nuclei (DAPI, blue) suggesting that the NPC culture is > 96% pure. Imaging was performed in Olympus confocal FV1000 microscope and processed with Adobe Photoshop CS4. Scale bar as noted.

Over 96% of control cells plated on poly-L-ornithine/laminin coated plates were immunoreactive for nestin at the end of treatment and 24 h later, confirming the purity of NPC culture (Fig 1).

### Propofol Does Not Cause NPC Death

**Fig 2:**
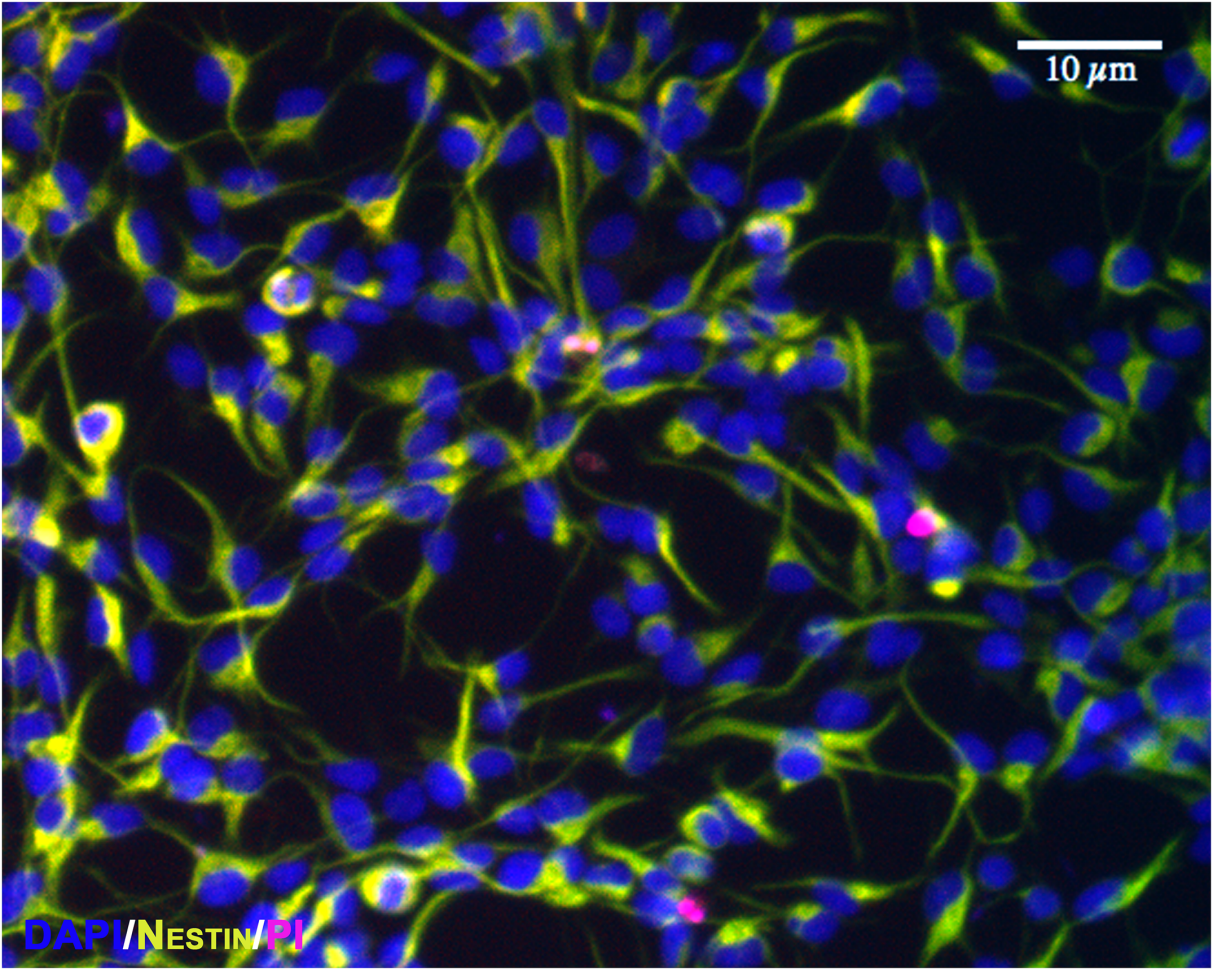
NPCs are healthy at baseline. A representative 20x image from a control well showing that the overall proportion of cell death, as measured by propidium iodide staining (pink), is less than 2% at baseline. Because of wavelength considerations, nestin was revealed with Alexa-Fluor^®^ 488 (green). Scale bar as noted.

**Fig 3:**
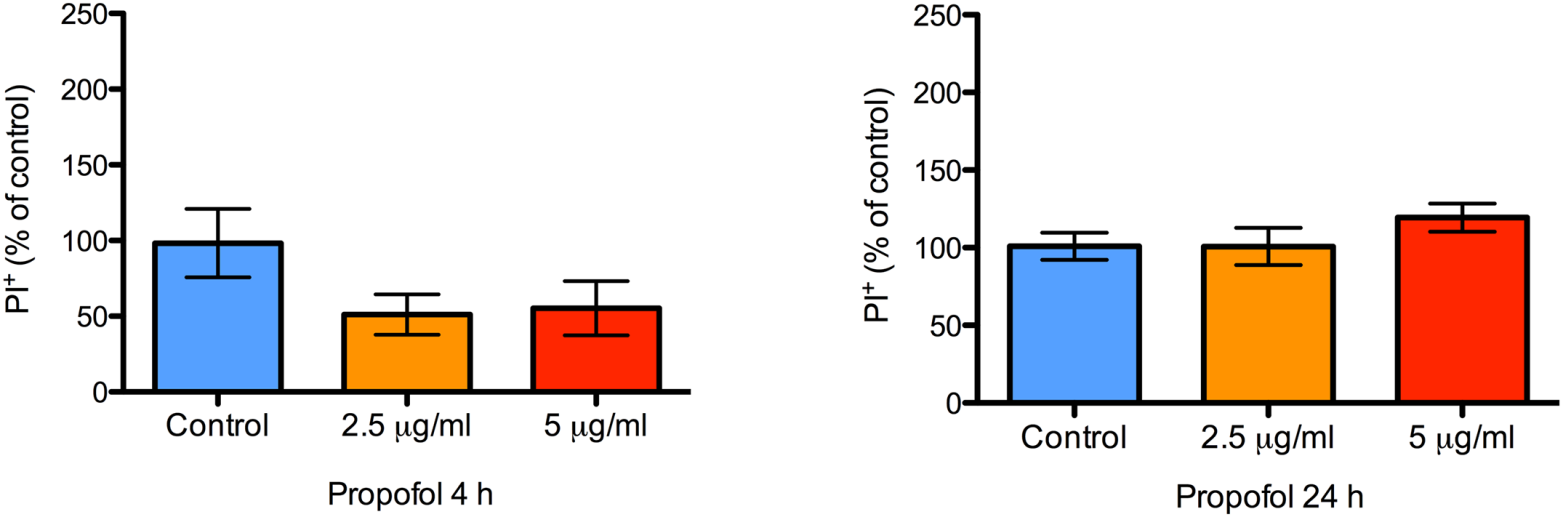
No difference in NPC death at the end of propofol treatment. A bar graph showing the proportion of dead NPCs in wells treated with 2.5 or 5 μg/mL of propofol for either 4 or 24 h as noted. Cell death was quantified with PI staining. There were no differences in NPC death at both concentrations compared to control treatment either at 4 or at 24 h. Data are presented as mean ± S.E.M and expressed as percent of the control value (N = 3 per condition; P = 0.08 and 0.32 at 4 and 24 h with Kruskal-Wallis test and 1-way ANOVA, respectively).

**Fig 4:**
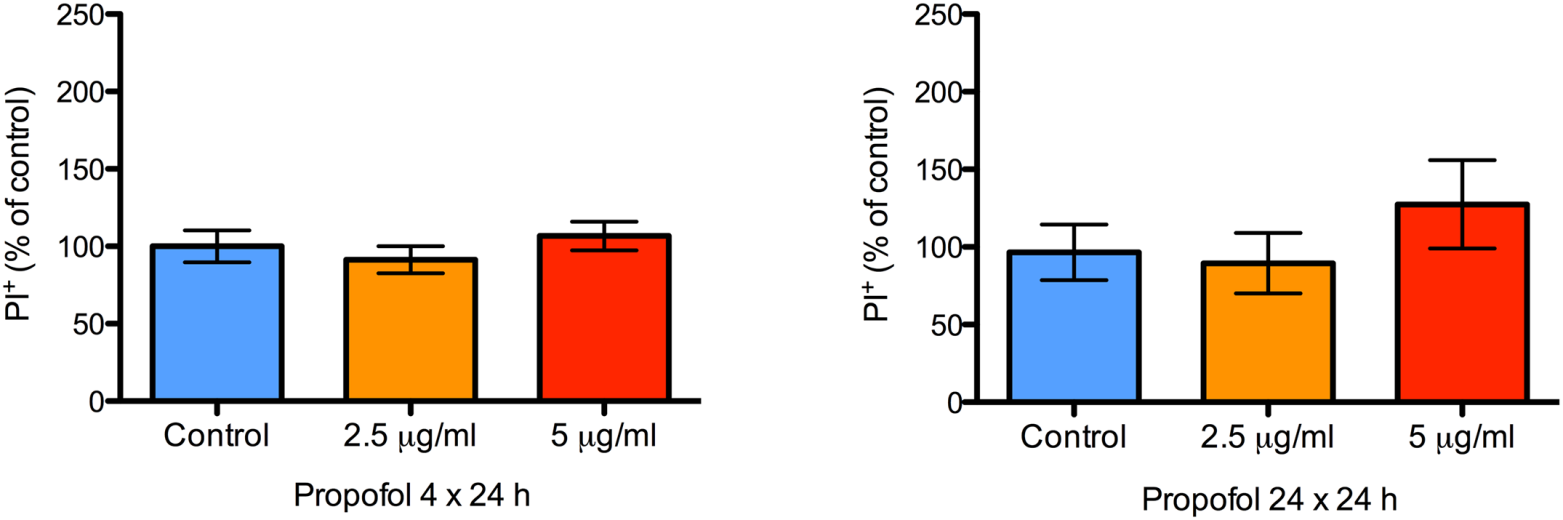
No difference in NPC death 24 h after discontinuation of propofol treatment. A bar graph showing the proportion of dead NPCs in wells treated with either 2.5 or 5 μg/mL of propofol, 24 h after discontinuation of treatment as noted. Cell death was quantified with PI staining. There were no differences in NPC death at both concentrations compared to control treatment 24 h after discontinuation of propofol. Data are presented as mean ± S.E.M and expressed as percent of the control value (N = 3 per condition; P = 0.52 and 0.45 for the 4 and 24 h treatment groups, respectively, with 1-way ANOVA).

PI is a cell impermeable dye that labels DNA only when cellular integrity is compromised and is widely used to measure of cell viability. There were few dead or dying cells, as determined by PI staining, at any time under control conditions (< 2%; Fig 2), indicating that the cultures were healthy. Similiarly, there was no effect of propofol treatment (both 4 and 24 h) on the percentage of cells that stained positive for PI at the end of treatment (P = 0.08 and 0.32, respectively; Fig 3) or 24 h after treatment (P = 0.52 and 0.45, respectively; Fig 4).

### Propofol Does Not Induce NPC Apoptosis

**Fig 5:**
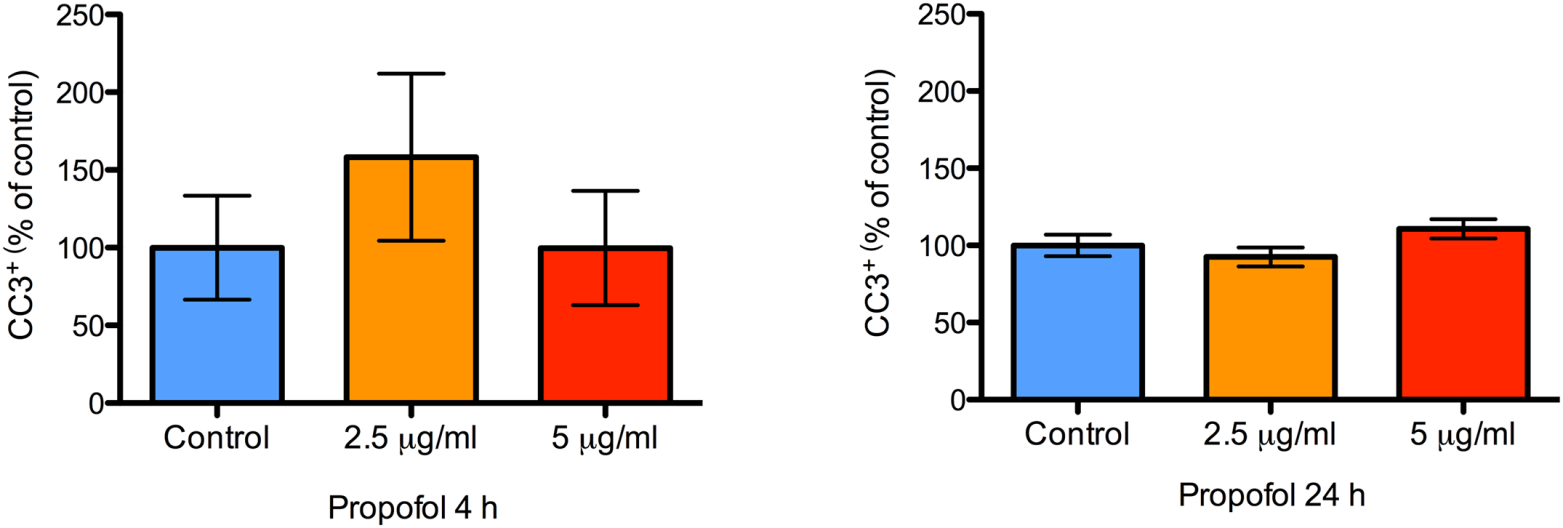
No difference in NPC apoptosis at the end of propofol treatment. A bar graph showing the proportion of apoptotic NPCs in wells treated with 2.5 or 5 μg/mL of propofol for either 4 or 24 h. Apoptosis was quantified with CC3 immunocytochemistry. There were no differences in NPC apoptosis at both concentrations compared to control treatment either at 4 or at 24 h. Data are presented as mean ± S.E.M and expressed as percent of the control value (N = 3 per condition; P = 0.51 and 0.14 at 4 and 24 h, with Kruskal-Wallis test and 1-way ANOVA respectively).

**Fig 6:**
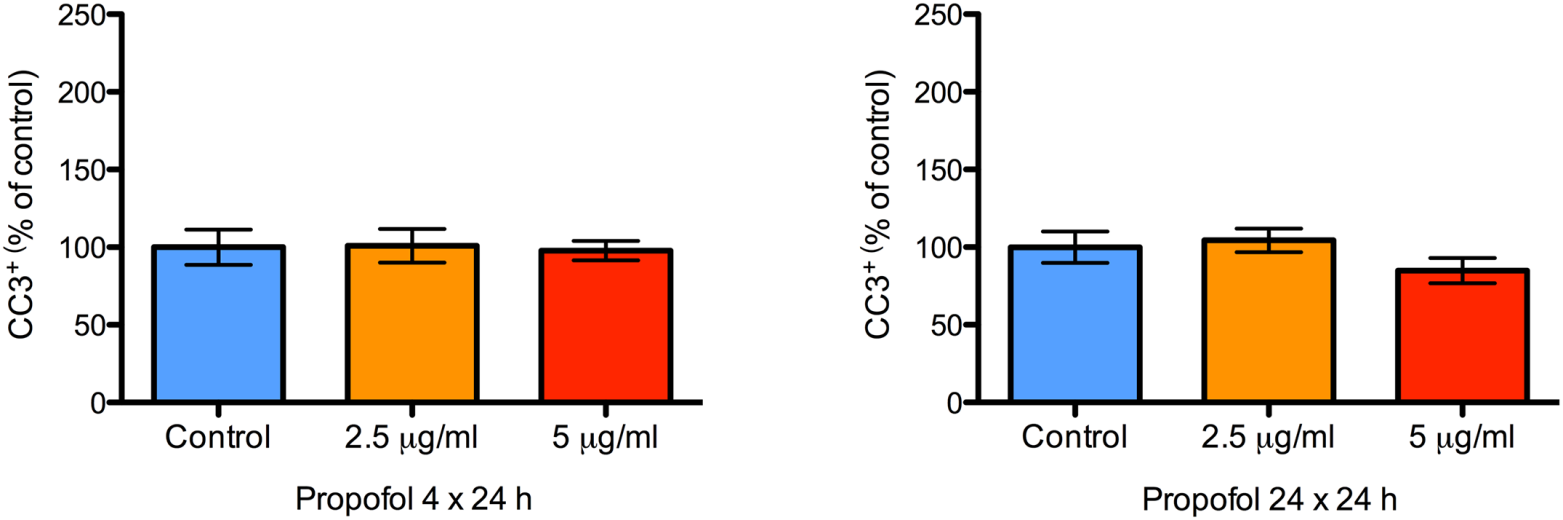
No difference in NPC apoptosis 24 h after discontinuation of propofol treatment. A bar graph showing the proportion of apoptotic NPCs in wells treated with either 2.5 or 5 μg/mL of propofol, 24 h after discontinuation of treatment. Apoptosis was quantified with CC3 immunocytochemistry. There were no differences in NPC apoptosis at both concentrations compared to control treatment 24 h after discontinuation of propofol. Data are presented as mean ± S.E.M and expressed as percent of the control value (N = 3 per condition; P = 0.61 and 0.24 for the 4 and 24 h treatment groups, with Kruskal-Wallis test and 1-way ANOVA respectively).

Cleaved caspase-3 is a critical component of apoptosis and is a marker for programmed cell death. Few cells stained positive for cleaved caspase-3 under control conditions (< 2%) and there was no effect of 4 or 24 h of propofol treatment on the percentage of cells staining positive for cleaved caspase-3 at the end of treatment (P = 0.51 and 0.14, respectively; Fig 5). 24 h after withdrawal, there were no differences in cleaved caspase-3 expression in the group that was treated with either 4 or 24 h of propofol (P = 0.61 and 0.24, respectively; Fig 6). Collectively, these data indicate that propofol has no cytotoxic effect on NPCs at the concentrations tested.

### Prolonged Treatment with Propofol Transiently Impairs Proliferation of NPCs

**Fig 7:**
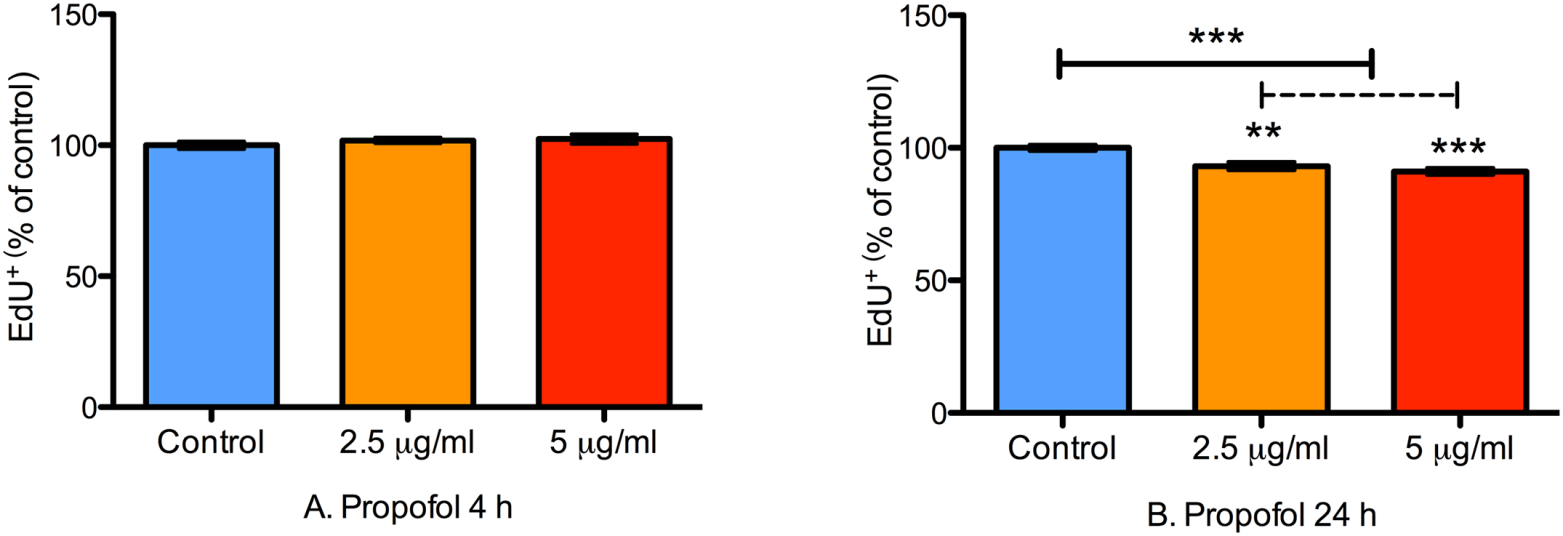
24 h of propofol treatment impairs NPC proliferation. A bar graph showing the proportion of proliferating NPCs in wells treated with 2.5 or 5 μg/mL of propofol for either 4 or 24 h. Proliferation was quantified with EdU immunocytochemistry. There were no differences in NPC proliferation at 4 h compared to control treatment (Fig 7A). However, both concentrations of propofol significantly decreased NPC proliferation in the 24 h treatment group (Fig 7B). Data are presented as mean ± S.E.M and expressed as percent of the control value (N = 3 per condition; P = 0.13 and < 0.0001 at 4 and 24 h, with Kruskal-Wallis test and 1-way ANOVA respectively); ** P ≤ 0.01 and *** P ≤ 0.001.

**Fig 8:**
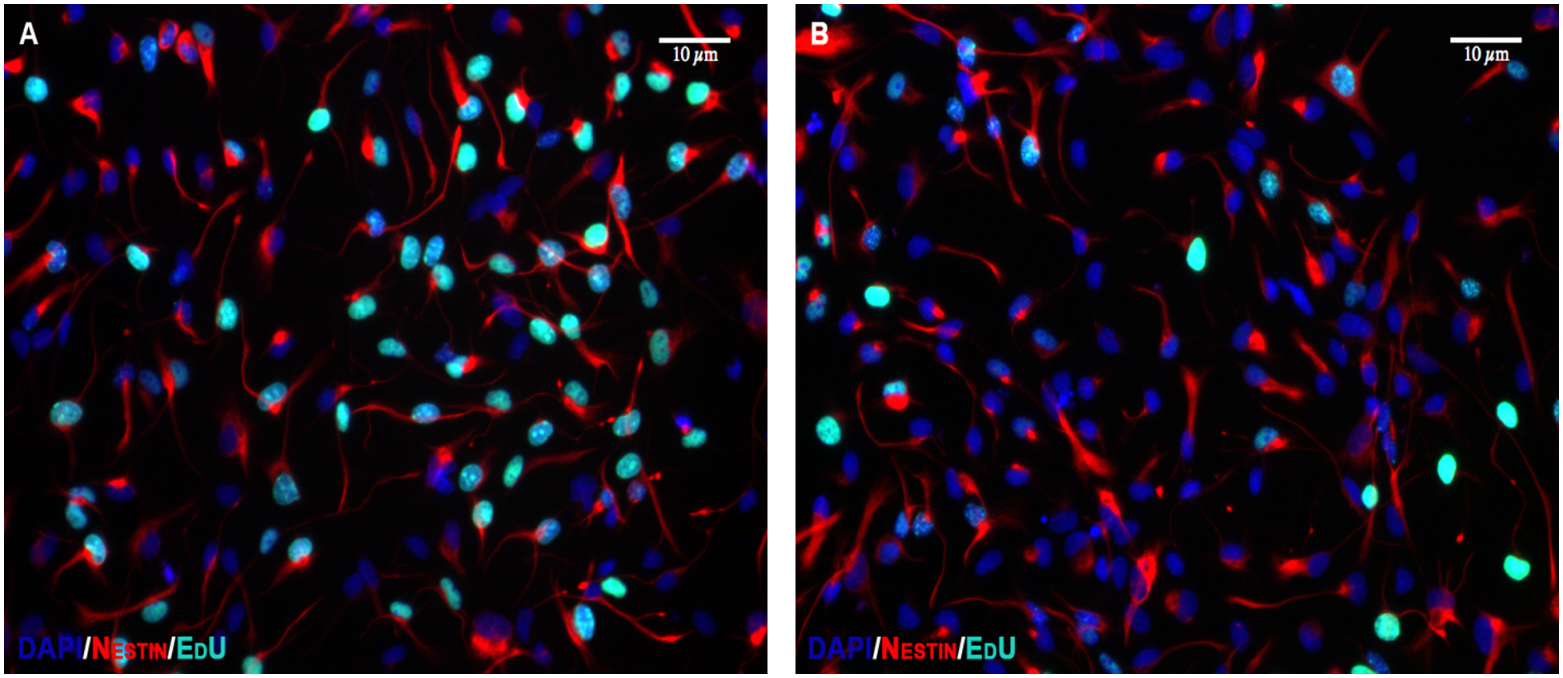
Effect of propofol on EdU incorporation at 24 h. Representative 20x photomicrographs of control (Fig 8A) and propofol-treated (Fig 8B) NPCs stained with nestin (red). 24 h of treatment with propofol causes a decrease in the number of NPCs that incorporate EdU (green) compared to control. Scale bar as noted.

Cell proliferation was determined with EdU incorporation. Like BrdU, EdU is a thymidine analogue that incorporates into actively mitotic cells during the S-phase of cell division and is widely used to assess cell proliferation [43]. The advantage of EdU is that it eliminates the need for the heating step associated with BrdU immunohistochemistry. Propofol impaired proliferation of NPCs in a duration of exposure-dependent manner as revealed by decreased EdU incorporation. With 4 h of treatment, both 2.5 and 5.0 μg/mL of propofol did not affect EdU incorporation in NPCs suggesting that 4 h of propofol exposure does not alter NPC proliferation in vitro (P = 0.13; Fig 7A). However, with 24 h of propofol treatment, both concentrations significantly reduced EdU incorporation compared to control treatment (P < 0.0001 for propofol vs. control; Fig 7B). Representative 20x photomicrographs from control and propofol-treated wells are shown in Fig 8.

**Fig 9:**
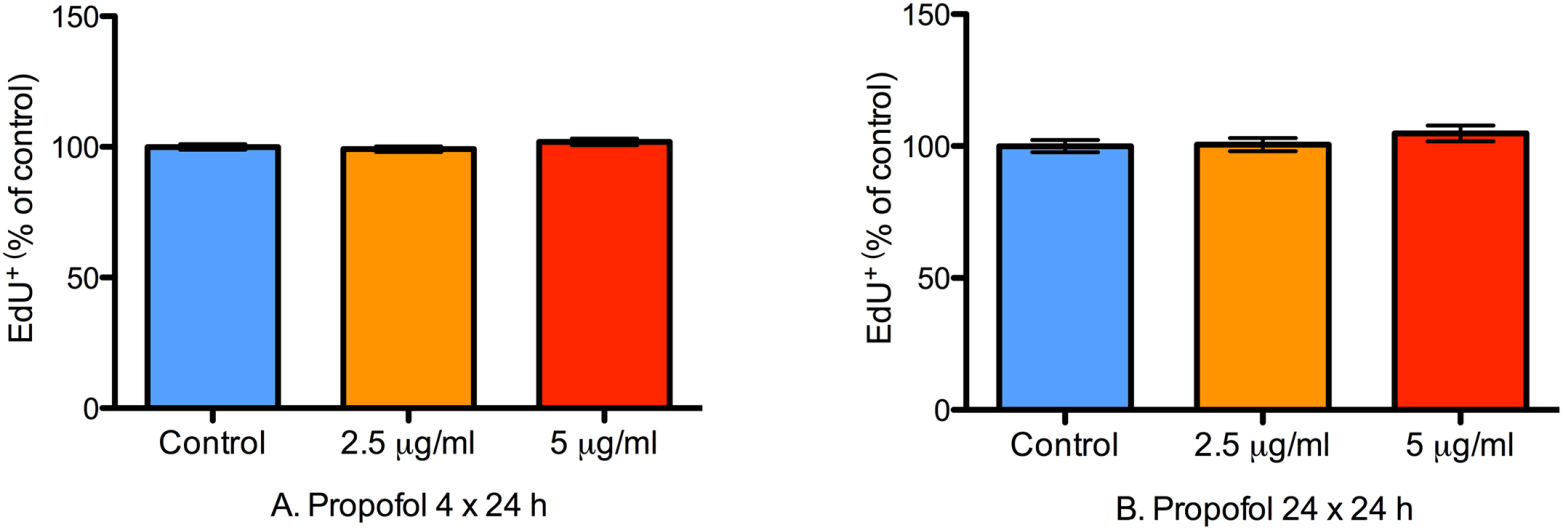
Propofol induced inhibition of NPC proliferation resolves within 24 h. A bar graph showing the proportion of proliferating NPCs 24 h after treatment with 2.5 or 5 μg/mL of propofol for either 4 or 24 h. Proliferation was quantified with EdU immunocytochemistry. There were no differences in NPC proliferation 24 h after treatment with both concentrations as measured by EdU incorporation. Data are presented as mean ± S.E.M and expressed as percent of the control value (N = 3 per condition; P = 0.21 and 0.38 for the 4 and 24 h treatment groups, respectively, with 1-way ANOVA).

To determine if these effects were transient, we studied EdU incorporation at 24 h following withdrawal of propofol in a separate set of experiments. After 24 h, there was no evidence of impaired EdU incorporation at either concentration of propofol (2.5 and 5.0 μg/ml) after either duration of treatment (4 h and 24 h; Fig 9A and 9B, respectively). These data collectively suggests that propofol transiently impairs NPC proliferation in a duration of exposure-dependent manner.

### Prolonged Treatment with Propofol Does Not Decrease Neural Progenitor/ Stem Cell Pool

**Fig 10:**
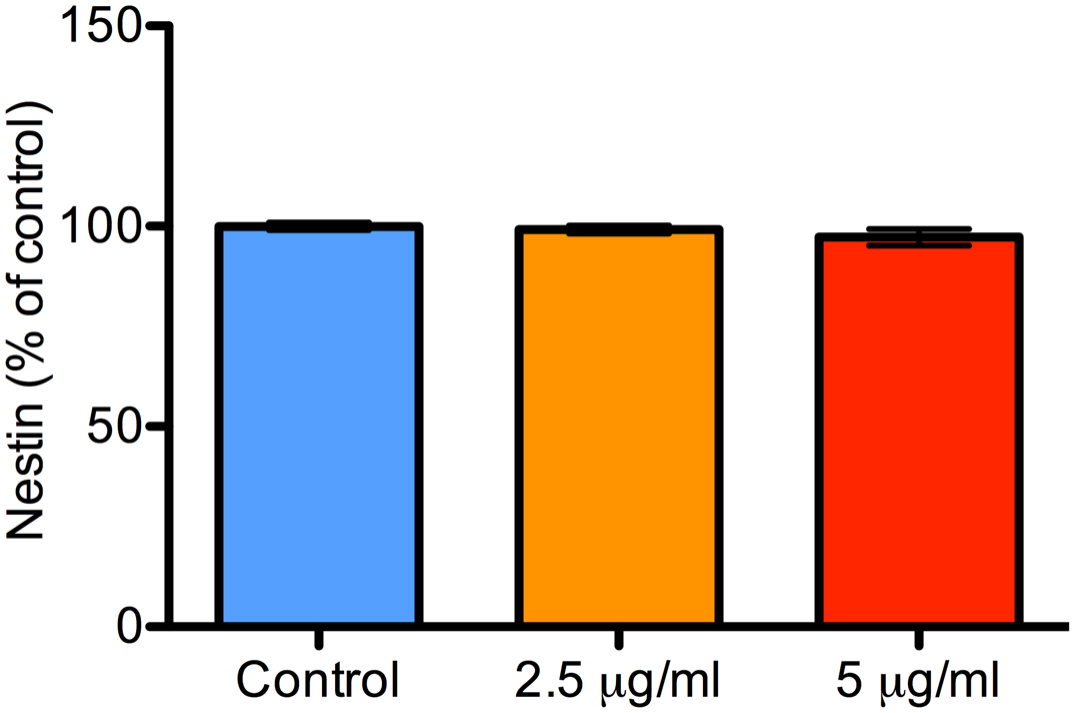
Prolonged treatment with propofol does not decrease the neural stem cell pool. A bar graph showing the proportion of NPCs 24 h after treatment with either 2.5 or 5 μg/mL of propofol for 24 h. NPCs were phenotyped with nestin immunocytochemistry. There were no differences in the overall proportion of nestin-positive NPCs 24 h after treatment with both concentrations. Data are presented as mean ± S.E.M and expressed as percent of the control value (N = 3 per condition; P = 0.67 with Kruskal-Wallis test).

Because we found a 7–14% overall decrease in the neural stem cell pool 24 h after isoflurane treatment,[25] we asked the question if propofol would induce a similar effect. To investigate this, we quantified the overall proportion of NPCs expressing the stemness marker nestin 24 h after propofol treatment (2.5 or 5.0 μg/mL for 24 h). There was no change in the overall proportion of nestin positive NPCs 24 h after both concentrations of propofol compared to control treatment (Fig 10). This suggests that, unlike isoflurane, propofol does not decrease the neural stem cell pool.

## Discussion

In this *in vitro* study, we present evidence that propofol impairs rodent NPC proliferation in a duration of exposure-dependent manner without affecting the viability of NPCs. Specifically, we found that treating NPCs with 2.5 or 5.0 μg/mL for 24 hours transiently impairs NPC proliferation which normalizes within 24 hours. Moreover, both concentrations of propofol had no effect on NPC survival at any time point. Finally, we also confirm that propofol treatment was not accompanied by a reduction in the overall neural stem cell pool. These findings contrast with our studies using isoflurane where the effect of treatment with isoflurane administered at or above 1 MAC concentration administered for 6 h lead to impaired NPC proliferation even 24 hours later with a reduction in the neural stem cell pool.[25] To our knowledge, this is the first in vitro study to investigate the impact of propofol on NPCs within the context of the concentration and duration of exposure ranges commonly observed in ICU practice.

Previous studies have focused on the effects of propofol administered during synaptogenesis, the phase of active brain growth during early development. Specifically, propofol administration was associated with robust neurodegeneration only in postnatal day 7–10 (PND 7–10) pups, but not at PND 14 or beyond, suggesting that the effect is developmental time-sensitive.[44, 45] Since synapse pruning and sculpting of neural circuits are largely completed during adolescence, these studies are less helpful in understanding the effects of propofol on the mature brain. Neurogenesis does continue in the mature brain, however, and multiple neurogenic niches exist in the subventricular zone, the subgranular zone, and the dentate gyrus. These niches contain NPCs that proliferate, migrate, and integrate into pre-existing neural circuits and play a large role in consolidation of learning, memory, and visual-spatial orientation.[11–15, 26, 46, 47] Because propofol is widely used for sedation in mechanically ventilated patients, it raises the interesting question if prolonged exposure to propofol at sub-anesthetic concentrations, as is typical for ICU sedation, affects NPC biology to the extent that it impairs cognitive function.

To begin to address this question, we sought to determine the effect of propofol on proliferation of NPCs at two different concentrations (2.5 and 5.0 μg/mL) and durations (4 and 24h). These concentrations of propofol were chosen after careful consideration of the predicted effect-site concentration of propofol at loss of consciousness.[38, 41, 48] We found that both 2.5 and 5.0 μg/mL of propofol inhibited NPC proliferation when administered for 24 h. The finding of decreased NPC proliferation after 24 h of propofol is consistent with the findings of Liu et al., [23] suggesting that the duration of propofol treatment is a critically important factor when clinically relevant concentrations are tested. More importantly, we confirmed that the impaired proliferation of NPCs is transient, because there was no change in EdU incorporation at 24 hours after discontinuation of propofol.

There was no evidence for NPC death at either concentration of propofol at any time point. This finding contrasts with other in vitro studies suggesting that propofol induces apoptosis and causes cell death in NPCs.[34–36] These differences can be readily reconciled when the concentrations of propofol are taken into consideration. Propofol-induced NPC apoptosis occurred only at supraclinical concentrations, i.e., 50 μM or higher, which is equivalent to approximately 8.9 μg/mL or greater (molecular weight of propofol = 178 g/moL). Pharmacokinetic studies with propofol show a consistent concentration-effect response with a plasma propofol concentration of 1.5–2.0 μg/mL correlating with deep sedation and 4.0–5.0 μg/mL correlating with general anesthesia, at least in adult humans.[40, 41, 49] For our experiments, we settled on the upper limit of the concentration range, i.e., 5.0 μg/ml of propofol. Our choice of 4 and 24 hours of propofol treatment to mirror total intravenous anesthesia and critical care sedation, respectively, was arbitrary but well within the acceptable range of clinical practice. Indeed, Sall et al. did not find evidence for NPC toxicity at a propofol concentration of < 7.1 μM (equivalent to ≈ 1.2 μg/mL).[50] Therefore, concentration is probably an important determinant of propofol neurotoxicity, with concentrations ≤ 5.0 μg/mL being unlikely to have any significant impact on NPC survival. The lack of cytotoxic effect of propofol is in contrast to the effect of propofol on immature neurons, where in vitro and in vivo studies show caspase activation and neuronal death.[51–54] We speculate this is because NPCs are at a relatively more primordial stage of development; they have GABA_A_ receptors [50] but may lack the full range of membrane receptors and subcellular signaling mechanisms that make immature neurons vulnerable to pharmacologic insults. Along these lines, isoflurane, which causes a 20–30% decrease in NPC proliferation,[25] does not kill NPCs, and ketamine, at a wide range of concentrations, likewise impairs NPC proliferation without causing cell death.[55] Thus, whatever the mechanism, NPCs appear to be inherently more resistant to pharmacological insults than immature neurons. Finally, prolonged treatment with propofol did not decrease the overall pool of NPCs. This contrasts with preclinical studies that show a decrease in the neural stem cell pool with isoflurane.[25, 28] We speculate that this is perhaps due to the modest and transient suppression of NPC proliferation, or due to differences in the mechanism of action between propofol and isoflurane [56, 57].

Our results are generally concordant with preclinical and clinical studies that have examined the effect of propofol on cognitive function, particularly in the adult brain. For example, a 2 h of propofol anesthesia in aged rodents (18 month old) does not affect hippocampus-dependent spatial memory,[58] and nor does a 3 h propofol anesthetic affect olfactory learning in 21-month-old aged rats. [30] Both the dentate gyrus of the hippocampus and the olfactory bulb are sites of robust adult neurogenesis.[59–61] In fact, in the multicenter BRAIN-ICU trial, sedation with benzodiazepines, but not propofol, was associated with impaired executive function 3 months later, though neither agent influenced global cognition scores.[3] This suggests that propofol is free of the significant long-term neurocognitive consequences that have been reported with other GABA_A_ agonists such as isoflurane, [62] perhaps in part because it does not diminish the pool of neural progenitor cells.

Our study is limited in some ways. First, as we had previously reported, EdU is a thymidine analogue that is incorporated into DNA only during the S-phase of cell division. Therefore, it is possible that we missed subtle alterations in the other phases of the cell cycle. Second, we arbitrarily chose to study the effects of propofol withdrawal after 24 h and therefore, it is possible that we may have missed immediate changes. However, the finding that the suppression of NPC proliferation is transient and resolves within 24 h argues against a toxic effect. Third, because critically ill patients are usually sedated with other agents in addition to propofol, we do not know whether a combination of such agents will have a more pronounced effect on NPC biology. Further, biological systems are inherently complex, so our *in vitro* results should be interpreted cautiously as multiple factors are likely to influence NPC biology in vivo, and studies show that mild, functional impairment can occur with propofol treatment via mechanisms unrelated to NPCs. In addition, we are unable to comment on the effect of propofol on NPCs beyond 24 h, a duration that is not uncommon during intensive care unit sedation. Finally, there is some uncertainty about the in vitro concentration of propofol that emulate those achieved clinically during sedation and anesthesia. This is because clinical studies typically measure blood concentrations of propofol, which may not accurately predict the concentration of the free, unbound, pharmacologically active fraction, and use multi-compartment mathematical models to calculate effect site concentrations that might not be relevant to single compartment in vitro studies. However, a rat microdialysis study showed that the free, unbound concentration of propofol in the brain interstitial fluid is approximately 50–60% of the measured total plasma level after a continuous infusion.[63, 64] This suggests the propofol concentrations and exposure conditions we used are clinically relevant, and that even concentrations consistent with deep propofol general anesthesia are without long term effects on NPC fate.

## Conclusions

In summary, our results demonstrate that propofol has 1) no effect on NPC survival in vitro; 2) no effect on NPC proliferation when exposure is limited to 4 h; and 3) an anti-proliferative effect when exposure lasts 24 h that fully resolves by 24 h after exposure. This suggests that prolonged treatment with propofol is unlikely to diminish the neural progenitor pool in vivo and that loss of neural progenitors is unlikely to explain neurocognitive dysfunction in ICU survivors sedated with this medication. However, whether propofol affects neurogenesis or cell fate selection remains to be determined.

## Supporting Information

S1 Dataset (PZF)
